# Postmortem redistribution of drugs: a literature review

**DOI:** 10.1007/s12024-023-00709-z

**Published:** 2023-09-16

**Authors:** Ghadeer M. M. Abdelaal, Nagah I. Hegazy, Rasha L. Etewa, Ghada E. A. Elmesallamy

**Affiliations:** 1 Department of Forensic Medicine and Clinical Toxicology, Faculty of Medicine, Zagazig University, Zagazig, Egypt; 2 Pathology Department, College of Medicine, Jouf University, Sakaka, Saudi Arabia

**Keywords:** Postmortem redistribution, Drugs, Cardiac blood, Ratio, Liver, Peripheral blood, Postmortem toxicology

## Abstract

Postmortem drug analysis is crucial in identifying the potential cause and manner of death. However, it is threatened by a significant phenomenon called postmortem redistribution (PMR), which refers to the alterations in drug levels occurring after death. This review aims to describe the PMR phenomenon, the mechanisms involved in the PMR of drugs, the various methods used to predict it, and various artifacts of postmortem drug concentrations. Several mechanisms, including passive diffusion from solid organs that act as drug reservoirs to the surrounding tissues, cadaveric changes after death (e.g., cell death, blood coagulation, hypostasis, and movements), and the putrefactive process, can result in artifacts of postmortem drug concentrations. The drug’s chemical and pharmacokinetic properties (such as acidic/basic properties, lipophilicity, protein binding, high volume of distribution, and residual metabolic activity) are additional factors. Several markers, including cardiac blood-to-peripheral blood ratio (C/P), liver-to-peripheral blood ratio (L/P), amino acid markers such as methionine, quantitative structure–activity relationship (QSAR) approach, and F factor, have been proposed for interpreting the liability of drugs to PMR. Several artifacts may affect the reliability of postmortem drug analysis. Peripheral blood is preferred for postmortem drug sample collection. Numerous laboratories evaluate the redistribution potential of drugs after death using the C/P concentration ratio. Nevertheless, the L/P concentration ratio is proposed to be a more reliable marker for PMR determination.

## Introduction

In forensic toxicology, drug testing differs from clinical toxicology testing. In cases involving forensic autopsies, drugs are examined in the context of death (postmortem toxicology) in various sample matrices. In contrast, clinical toxicology focuses on drug monitoring in both acute and chronic toxicities, and specimens are primarily confined to urine and blood [1].

During postmortem forensic investigation, the forensic toxicologist seeks to identify any legal or illegal drug use prior to death and assess its contribution to the cause of death. The determining factor in this situation is whether the concentration in a postmortem sample accurately reflects the concentration at the time of death. Postmortem redistribution (PMR) describes anatomical and physiological changes that can falsely alter concentrations after death [2].

Cardiac blood is more susceptible than peripheral blood to PMR shifts. Therefore, the ratio of cardiac blood to peripheral blood concentration (C/P) is a commonly used marker for the prediction of PMR of drugs in forensic autopsies [3].

In the first section of this literature review, we provide an overview of the PMR phenomenon. In the next section, we explain the mechanisms involved in the PMR of drugs. In the third section, we discuss the different approaches used in the prediction of PMR for drugs. Finally, in the fourth section, we discuss the various artifacts of postmortem drug concentrations.

Several procedures were followed to ensure a high-quality review of the literature on PMR of drugs. A comprehensive search of peer-reviewed journals was completed based on a wide range of key terms including, postmortem redistribution, postmortem drug analysis artifact, cardiac blood to peripheral blood ratio, liver to peripheral blood ratio, and redistribution amino acids markers. Five databases were searched, including Elsevier, Springer, Google Scholar, PubMed, and Wiley Online Library. Also, the reference section for each article found was searched in order to find additional relevant articles. The search process uncovered 22 peer-reviewed articles published from 2003 to 2023.

## Postmortem redistribution (PMR)

For several decades it has been known that postmortem drug concentration can differ considerably. Gee et al., 1974 were the first to report PMR and then many research works have been done to prove the occurrence of such a phenomenon. According to postmortem interval, environment, sampling site, volume of distribution, redistribution to body cavities, and degradation after death, drug concentrations within the corpus may vary [4, 5].

The interpretation of postmortem toxicology results is challenging, owing to changes that occur within the body after death. Significant alterations in blood drug concentrations can occur due to postmortem drug movement and instability. Therefore, PMR has gained its reputation a “toxicological nightmare” [6].

In autopsy cases, the quantification of drugs in samples obtained from the deceased is essential for determining the potential cause of death, whether intoxication, adverse effects, or lack of compliance with medical therapy. Peripheral blood is regarded as the gold standard for measuring postmortem drug concentrations [7].

Despite the fact that postmortem drug concentrations may not always correspond to premortem levels, they may follow recognizable patterns that facilitate interpretation. For instance, the characteristics of the drug itself can predict how likely it is to be redistributed after death. For example, basic lipophilic drugs with a volume of distribution higher than 3 L/kg are likely to undergo PMR. Blood drawn from the heart and the center of the body contains higher concentrations of these substances than blood drawn from peripheral vessels. One possible explanation for this phenomenon is the diffusion of these drugs from solid organs such as the liver, lungs, and heart [8].

It is possible to assess the redistribution of drugs after death by comparing postmortem blood concentrations with antemortem specimens, which are rarely available outside of hospital deaths. Consequently, PMR susceptibility is typically determined by the central blood-to-peripheral blood ratio (C/P). A different approach, the liver-to-peripheral blood ratio (L/P), has also been proposed. Although sampling protocols for toxicological postmortem examinations have not yet been universally standardized, central blood, peripheral blood, and liver are frequently collected for the determination of C/P and L/P ratios [6].

## Mechanisms of PMR of drugs

McIntyre [9] illustrated that the mechanisms underlying PMR are very sophisticated and still not entirely understood. However, postmortem drug concentrations in the blood can follow certain generally approved patterns that may aid in the interpretation of their analysis.

Pélissier-Alicot et al. [10] categorized the main mechanisms that may explain PMR of substances into passive diffusion from drug reservoirs (e.g., gastrointestinal tract, lungs, heart, and liver), cadaveric changes (e.g., cellular death, blood hypostasis, blood movements, and putrefactive process) and the chemical and pharmacokinetic features of the drug (e.g., pKa, lipophilicity, protein binding, the volume of distribution, and postmortem residual metabolic activity) (Table 1).

**Table 1:** Mechanisms for postmortem redistribution and their consequences [10]

**Mechanisms**	**Consequences**
**Drug reservoirs:**	**Redistribution to surrounding tissues:**
- Gastrointestinal tract	Cardiac chambers, thoracic vessels, left lung, liver, inferior vena cava.
- Lungs	Cardiac chambers, thoracic vessels, liver.
- Myocardium	Heart blood.
- Liver	Inferior vena cava, right cardiac chambers, pulmonary vessels, stomach, duodenum, gall bladder.
**Cadaveric changes:**	
- Cell death	Leakage of xenobiotics into the extracellular space
- Blood coagulation and hypostasis	Modification of serum/blood ratio
- Blood movements	Transport of xenobiotics and mixing of bloods from different origins
- Putrefactive process (bacteria)	Degradation and/or synthesis of macromolecules
**Drug chemical and pharmacokinetic properties:**	
- Acidic/basic properties	*
- Lipophilicity	*
- Drug binding proteins or red cells	*
- High volume of distribution	Leakage from tissues
- Residual metabolic activity	*

*Vary according to the drug properties

### Passive diffusion from reservoir organs

In the thoracic and abdominal cavities, drug reservoirs include organs with high concentrating capacity (liver, lung, and heart) and hollow viscera (e.g., various parts of the gastrointestinal tract). Due to the close proximity of these organs and the adjacent blood vessels, passive drug diffusion from reservoir organs is believed to be the primary source of drug PMR. Concentration gradients are produced by the unequal distribution of drugs within the body during life and by substances that are not completely absorbed by the digestive tract [11].

Redistribution from organs occurs either by diffusion through blood vessels or transparietal diffusion. Unabsorbed drugs in the stomach and airway contamination from drugs regurgitated at the time of death result in PMR. Weak lipophilic bases with pKa > 8 are susceptible to lung sequestration. Lung redistribution is more intense than redistribution from the gastrointestinal tract or liver due to its large alveolar surface area, thin diffusion membrane, and high vascularization. Heart-sequestered drugs rapidly diffuse to cardiac blood evenly. However, higher drug concentrations in cardiac blood than in the myocardium indicate redistribution from the lungs. Drugs concentrated in the liver diffuse mainly through hepatic veins towards either the inferior vena cava to the right cardiac chamber or peripheral venous blood, another less significant way is direct diffusion to the stomach and gall bladder [10].

The redistribution of drugs primarily occurs in the first postmortem hours prior to the stage of putrefaction [12]. Passive diffusion from reservoir organs has been suggested to be the main mechanism involved in this early postmortem period for many substances [13].

The tendency of drugs to accumulate in reservoir organs during life depends on their physicochemical properties. Due to diffusion from organs with high concentrations, their concentration in the central blood after death may be elevated. Peripheral blood samples are anatomically isolated from the thoracic cavity and are considered more indicative of antemortem blood drug concentrations [6].

In some autopsy cases, it is difficult to obtain peripheral blood due to advanced degree of decomposition, severe burn, or massive hemorrhage. In such situations, other matrices (e.g., vitreous humor and muscles) are collected. However, for most drugs, there is a lack or even absence of information required for interpretation and comparing concentrations in different matrices [7].

### Cadaveric changes

Following clinical death, cellular ischemia results in cell death and the release of its contents, including drugs (i.e., oxidative phosphorylation ceases, ATP synthesis decreases, and anaerobic metabolism begins). Due to the accumulation of lactic acid and inorganic phosphates, the intracellular pH drops, and the Na-K ATPase pump fails secondary to decreased ATP production. Within the cell, sodium accumulation causes cellular edema and endoplasmic reticulum dilatation. Mitochondrial matrix and lysosomal membranes are destroyed with leakage of their enzymes into the cytoplasm. Finally, cell components are degraded and released into the extracellular space [14].

Regarding biochemical changes that occur immediately after death, the failure of the Na-K ATPase pump causes a rapid rise in potassium to significantly high concentrations even before hemolysis. Cell membrane integrity is lost, and various complex mechanisms of xenobiotic transport, metabolism, and storage within cells and inside vesicles fail over variable postmortem time intervals. However, the environment in which the body is stored and the amount of time that has passed since death have a significant impact on drug concentrations [15].

The occurrence of cellular acidification and autolysis can cause the accumulation of basic substances in tissues and decrease the protein-binding properties of some compounds. Cellular autolysis and bacterial metabolism have been suggested to cause alteration in concentrations of drugs in both central and peripheral sites in the late postmortem period [13].

Blood hypostasis and coagulation occur unevenly throughout the body. A large number of erythrocytes are entrapped by blood clots, and the degree of their lysis determines blood fluidity and movement. If a blood clot is obtained during blood sampling for toxicological testing, concentrations of drugs with unequal distribution between serum and erythrocytes will be inaccurately measured. Additionally, blood movement within vessels can occur in the early postmortem period due to the effect of rigor mortis, which induces ventricular contraction with minimal blood flow from the heart into the neck veins. In addition, the increased intra-abdominal pressure causes blood reflux from the abdominal aorta into the thoracic aorta, from the inferior vena cava into the right atrium, and from the left cardiac side into the pulmonary veins. This movement is influenced by pressure and fluidity changes and can be responsible for the physical redistribution of drugs within vascular compartments [10].

Changes in drug concentrations at various anatomical sites may also be influenced by postmortem blood movement dependent on gravity. In an animal model, for instance, a drug diffuses primarily from the stomach to the left lobe of the liver. This is of little consequence when specimens are collected from peripheral sites, and the effect of body size may alter its significance (small animal size versus the larger adult human body). Furthermore, some studies have revealed that when bodies are left in a supine position, the postmortem diffusion of basic drugs in the lungs occurs rapidly into the left heart chambers through the pulmonary venous blood flow. In addition, basic drugs (cardiac glycosides, local anesthetics, opioids, and tricyclic antidepressants) tend to accumulate in the myocardium, and diffusion into heart blood after death may lead to an elevation in heart blood concentrations [16].

Bacterial invasion of the corpus starts almost immediately after death and metabolizes many drugs (e.g., sulfur-containing antipsychotics and nitrobenzodiazepines). In addition, ethanol can rise to high concentrations due to bacterial metabolism, which is of significant importance in investigating traffic accidents [15].

### Drug properties

Although almost all drugs exhibit some degree of PMR, lipophilic, basic, highly protein-bound drugs and those with a high volume of distribution (Vd) are particularly susceptible to this phenomenon [13].

Lipophilic drugs and organic bases tend to accumulate in solid organs (e.g., lungs, liver, and heart). This creates a concentration gradient that favors postmortem passive diffusion. Upon cell death and the subsequent decrease of intracellular pH, basic drugs become more ionized, and after cell lysis, they distribute more readily. Moreover, drugs with a Vd greater than 3 L/kg are widely distributed in tissues and have the greatest potential for PMR because they are released into plasma after death, resulting in a rise in their blood concentrations [14].

There is a reduction of the total protein blood concentration after death due to acute anoxia that leads to the breakdown of proteins into amino acids and peptides, as well as proteolysis by auto enzymes. Proteolytic bacteria accelerate the process during putrefaction. In addition, serum albumin is reportedly susceptible to PMR in the perivascular space, and protein binding capacity decreases rapidly. Consequently, concentrations of drugs in their free form are elevated in the intravascular space after death [10]. However, caution must be exercised when comparing whole blood and plasma drug concentrations for a predominantly protein-bound drug, as whole blood and plasma concentrations may vary significantly [17].

Postmortem residual metabolic activities within the body continue the transformation of many drugs, such as cocaine, which is lowered by hydrolysis to benzoylecgonine, besides its enzymatic conversion to ecgonine methyl ester. Additionally, continuing metabolic activities increase the concentrations of some physiological substances, such as gamma-hydroxybutyrate [15].

## Markers of PMR of drugs

### Cardiac blood to peripheral blood ratio (C/P)

The C/P ratio of drug concentrations in postmortem blood has been used to assess a compound’s probability of undergoing PMR. Drugs with high ratios are supposed to have a greater propensity for redistribution (Table 2). In such cases, the allocation of autopsy samples from either a cardiac chamber or the pericardium alone may result in the measurement of a drug concentration that is considerably higher than the peripheral concentrations, leading to an erroneous interpretation of the results [16].

**Table 2:** List of compounds with propensity for postmortem redistribution using cardiac blood to peripheral blood ratio (C/P) [15]

Alprazolam (1.5)	Methamphetamine (2.4)
Amantadine (2)	Methylenedioxy methamphetamine or MDMA (2.65)
Amitriptyline (3.1)	Methyprylon (1.9)
Amoxapine (1.8)	Metoprolol (3.8)
Amphetamine (2.0)	Mexiletine (3.6)
Benztropine (1.3)	Midazolam (4)
Bupropion (1.9)	Morphine (heroin) (2.2)
Chloroquine (3.0)	Naproxen (1.5)
Chlorpheniramine (3.1)	N-methylbenzodioxazolybutamine MBDB (2.5)
Chlorpromazine (4.0)	Nortriptyline (2.4)
Clomipramine (1.9)	Orphenadrine (1.9)
Clonazepam (2.0)	Oxazepam (1.3)
Clozapine (2.8)	Oxycodone (3.1)
Cocaine (1.5 to 2.3)	Paramethoxyamphetamine (1.6)
Codeine (1.8)	Paroxetine (2.7)
Cyanide (1.3)	Pentazocine (2)
Cyclobenzaprine (2.2)	Pethidine or meperidine (2.1)
D-methamphetamine (2.1)	Phencyclidine (1.8)
Desipramine (2.4)	Phentermine (1.7)
Dextromethorphan (2)	Phenylbutazone (2.3)
Diazepam (1.6)	Phenylpropanolamine (2.4)
Diltiazem (2.6)	Phenytoin (1.4)
Diphenhydramine (2.3)	Promethazine (1.6)
Dothiepin (3)	Propafenone (2.4)
Doxepin (5.5)	Propoxyphene (3.5)
Flecanide (3.6)	Propranolol (2.5)
Flunitrazepam (3)	Pseudoephedrine (1.5)
Fluoxetine (2.9)	Secobarbital (1.5)
Flurazepan (3.0)	Strychnine (15)
Fluvoxamine (1.7)	Temazepam (1.6)
Furosemide (1.7)	Thiopental (1.9)
Gamma hydroxybutyrate (2)	Timolol (2)
Haloperidol (3.6)	Tranylcypromine (2.2)
Hydrochlorthiazide (23)	Trazadone (1.6)
Ibuprofen (1.8)	Triazolam (2.8)
Imipramine (1.8 to 2.2)	Trichlorethanol (2.0)
Indomethacin (1.9)	Trimipramine (1.6)
Ketamine (1.6)	Venlafaxine (1.6)
Maprotiline (4.7)	Venlafaxine (1.6)
Meprobamate (1.7)	Zolpidem (2.1)
Mesoridazine (1.3)	

In an early trial to demonstrate PMR, postmortem blood samples were allocated from two sites at autopsy: peripheral and central (often the cardiac). This approach appeared to provide only a partial explanation for postmortem drug concentrations. However, the C/P ratio has become an accepted marker for PMR, with ratios > 1.0 indicating redistribution [9].

Although there are some practical applications, there are limitations to the C/P model. It is known that drug properties influence PMR, but no relationship has been established between the C/P ratio and drug properties. In addition, there has been little agreement regarding the ratio that detects the potential for significant, intermediate, or minimal redistribution of a substance. Furthermore, some studies reported C/P ratios > 1.0 for drugs known to be not subjected to PMR such as salicylate and tramadol. A C/P ratio > 1.0 in drugs that do not redistribute can result from statistical chance, arterio-venous differences, and anatomic variability between individuals. In addition, inaccurate ratios may also be due to an artifact of sampling when the heart blood is depleted by the collection of blood from the connected vessels, incomplete absorption/distribution in acute overdoses, and resuscitation attempts that may lead to a C/P ratio < 1.0. Hence, C/P ratios are inconclusive regarding the interpretation of PMR [17].

### Liver to peripheral blood ratio (L/P)

Although many laboratories evaluate drug redistribution in postmortem cases by C/P ratio, the L/P ratio is considered a more reliable alternative marker. Since postmortem drug concentration is more stable in tissues, the confidence of measuring drugs in blood after death is decreased, and the significance of tissue analysis comes back on the agenda. As an alternative to the C/P ratio, the L/P ratio has been proposed as a marker for PMR, with ratios < 5 indicating little to no propensity for redistribution and ratios > 20–30 indicative of PMR [15, 18].

McIntyre [17] studied the PMR of thirteen drugs and concluded that the L/P ratio surpassed the C/P ratio for every drug. He also concluded that there was no significant mathematical relationship between the L/P and C/P ratios. He observed that liver drug concentrations were often higher than those in the blood, providing an opportunity to evaluate those medications with a moderate degree of PMR. In contrast, the conventional C/P ratio model could not assess the degree of PMR in drugs as it yielded comparatively analogous ratios among different compounds (Table 3).

**Table 3:** Liver to peripheral blood ratios (L/P) and cardiac blood to peripheral blood ratios (C/P) (listed in order of increasing L/P ratio) [16]

**Drug**	**L/P (mean)**	**C/P (mean)**
Tramadol	1.6	1.1
Carisoprodol	2.8	1.3
Venlafaxine	5.0	1.3
Mirtazapine	5.8	1.1
Methadone	6.8	1.3
Lamotrigine	8.6	1.0
Quetiapine	9.0	1.4
Citalopram	9.9	1.2
Paroxetine	22	2.0
Olanzapine	23	1.3
Amitriptyline	25	3.0
Clomipramine	58	1.9
Sertraline	97	1.3

L/P ratios > 20 indicates postmortem redistribution

C/P ratios > 1 indicates postmortem redistribution

### Other PMR markers

Amino acids are suggested to be a valuable marker for PMR from the lungs. In particular, methionine revealed a positive correlation with xenobiotic concentrations from the pulmonary vasculature. Despite this correlation, this approach has not become common practice in forensic laboratories [14].

Quantitative structure–activity relationship (QSAR) methodology has been proposed to determine the probability of drugs undergoing PMR. This computer modeling application had limited success, but this technique may have potential in the future [19].

Giaginis et al. [20] applied QSAR in an attempt to model PMR data for benzodiazepines and tricyclic antidepressants. This approach employs many physicochemical and molecular properties of the drugs, such as molecular size, basicity, lipophilicity, and ionization energy. Despite the reasonable correlation achieved, the number of parameters applied did not provide sufficient accuracy to make this model reliable for predicting the degree of PMR.

McIntyre has added an “F” factor to the L/P ratio model to measure the potential of drugs to cause PMR. This factor is intended to demonstrate a direct correlation between postmortem peripheral blood concentration and antemortem whole-blood concentration (antemortem concentration = postmortem concentration/F). Currently, it is not easy to apply this factor in common practice. It needs the acquisition of a larger database than is available nowadays, as the measurement of liver concentrations is subjected to difficulties such as matrix effects [17].

McIntyre has also proposed a “theoretical” PMR factor (Ft) based on drug properties and L/P ratio concepts, with the unique L/P ratio of a drug being the only independent variable. This model was intended to facilitate a rational and credible interpretation of the analysis of postmortem drug concentrations by ranking drug’s potential for the expected degree of PMR [21].

## Artifacts of postmortem drug concentrations

Initially, using antemortem concentration for comparison was predicated on the assumption that the concentration of xenobiotics in the specimen would not change during or after death. This hypothesis was incorrect for a large number of compounds. Consequently, the tendency for changes in postmortem concentrations must be considered for all xenobiotics, with the exception of a few substances for which it has been demonstrated that PMR does not occur (Table 4) [16].

**Table 4:** Compounds in which postmortem redistribution probably does not occur [15]

Alcohols^a^ Carbon monoxide Carbamazepine Chlordiazepoxide Diflunisal Ephedrine Hydrocodone Hydroxyzine Lorazepam Lamotrigine	Mirtazapine Nitrazepam Phenelzine Pheniramine Phenobarbital Primidone Procyclidine Quinine/Quinidine Theophylline Zopiclone

^a^Ethanol concentration may rise in decomposed bodies due to postmortem fermentation

Postmortem concentration changes are primarily affected by the time passed since death, the route of drug administration, and the body’s position. Consequently, postmortem blood samples should be allocated as soon as possible. Femoral blood is generally preferred because it is less susceptible to PMR than central blood and is more abundant than other peripheral blood sampling sites. There are considerable differences between forensic medicine departments regarding sampling procedures, so standardization for postmortem blood sample collection has been recommended. Moreover, arteriovenous differences have been established in vivo and can also exist postmortem [11].

Postmortem storage temperatures can significantly alter drug concentrations. For instance, cocaine is more likely to be metabolized in a warm, alkaline environment, so its metabolism continues after death. Pathologists prevent metabolism inside blood samples by storing them at 4 °C and adding sodium fluoride. In addition, postmortem agonal aspiration may influence drug redistribution, which is of particular importance in analyzing cardiac drug concentrations as they may be falsely increased due to diffusion from adjacent lungs [14].

Although autopsy material is typically used to reveal the cause of death, toxicology testing often provides more data. However, drugs that are often detected via screening methods are usually irrelevant to the cause of mortality, e.g., a continual therapeutic medication, perioperative drug administration, or unsuccessful emergency therapy [22].

Not only redistribution but also postmortem degradation of xenobiotics can result in changes in drug concentration that may be misinterpreted as redistribution; for example, the hydrolysis of morphine glucuronides with prolonged postmortem intervals, as well as the postmortem bacterial metabolism of nitrobenzodiazepines [13].

Yarema and Becker [14] provided a summary of the questions a medical toxicologist should ask when analyzing postmortem drug concentrations. These questions are as follows: (1) What site was used for the blood sample? (2) If the femoral vein was used, was the vein ligated or clamped prior to sampling? (3) Was more than one site used for sampling? (4) Were tissues other than blood used for sampling (e.g., vitreous, lung, liver, skeletal muscle)? (5) How long after death was the sample taken? (6) How was the body stored in the interval between death and blood sampling? (7) Is there evidence of significant decomposition in the body? (8) Under what conditions was the blood sample collected and stored? (9) How long was the delay between when the sample was collected and when it was analyzed? (10) Is there any antemortem or perimortem clinical information available on the deceased? (11) Is there any antemortem or perimortem blood available for analysis? (12) What are the properties of the drug involved (pKa, lipophilicity, Vd)?

## Conclusion

Drug concentrations in peripheral blood after death are considered to be fairly comparable to those before death. Consequently, blood samples collected from peripheral blood are preferred for postmortem drug testing. Many laboratories use the cardiac blood-to-peripheral blood (C/P) concentration ratio to evaluate the redistribution of drugs in postmortem autopsies. However, the liver-to-peripheral blood (L/P) concentration ratio is proposed as a more reliable marker for estimating PMR since postmortem drug concentration is more stable in tissues.

## Key points

1. Postmortem redistribution is a complex phenomenon that can affect the interpretation of toxicology results.
2. Different mechanisms result in postmortem drug redistribution, such as passive diffusion from reservoir organs, cadaveric changes, and the liability of the drug to redistribute due to its chemical and pharmacokinetic properties.
3. There are various markers for the prediction of the redistribution of drugs, such as the C/P ratio, the L/P ratio, amino acid markers, and the QSAR approach.
4. Artifacts of postmortem drug concentrations can result from various factors, such as the time passed since death and sample collection, the sampling site, the storage temperature, and the drug properties.

## Data Availability

The data that support the findings of this study are available on request from the corresponding author.
